# Effects of virtual reality-based disaster simulation education on nursing students

**DOI:** 10.1371/journal.pone.0329563

**Published:** 2025-10-07

**Authors:** Kyeng-Jin Kim, Moon-Ji Choi, MinJi Kim

**Affiliations:** 1 College of Nursing, Kyungpook National University, Daegu, South Korea; 2 College of Nursing, Kyungil University, Gyeongsan, South Korea; University of Hong Kong, HONG KONG

## Abstract

**Background:**

This study aimed to confirm the effectiveness of disaster simulation education that uses virtual reality (VR).

**Method:**

This quasi-experimental study was conducted using a non-equivalent control group pretest-posttest design. Participants in this study were third- and fourth-year nursing students at two universities. A total of 67 nursing students were included in the analysis, with 33 in the experimental group and 34 in the control group. The experimental group underwent VR disaster simulation education based on cognitive continuum theory. The control group underwent written case simulation.

**Results:**

Disaster triage accuracy (t = 6.11, **p* *< .001), disaster triage confidence (t = 2.69, **p* *= .009), learning immersion (t = 6.88, **p* *< .001), and cognitive flexibility (t = 5.47, **p* *< .001) significantly increased in the experimental group.

**Conclusion:**

The possibility of using the VR simulation program based on cognitive continuum theory developed through this study should be further explored. An effective educational strategy is presented to strengthen prospective health care professionals’ disaster capabilities through VR disaster simulation education training.

## Introduction

Disasters are unpredictable and can significantly disrupt people’s daily lives in an instant. The unpredictability and potential for multiple casualties at disaster sites can challenge healthcare professionals serving as first responders [1]. Mass casualty events can lead to a shortage of medical resources, making it impossible to provide the usual level of medical services to everyone, which may increase morbidity and mortality rates [1,2]. Therefore, healthcare professionals at disaster sites must quickly and accurately identify the severity of the injuries of those affected and nature of the disaster [2].

In mass casualty incidents, triage systems use medical criteria to evaluate the condition of patients and assign triage levels [3]. The World Health Organization and International Nursing Council emphasize the role of nurses as first responders in on-site disaster triage [3,4]. However, studies have shown that nurses often have insufficient knowledge and coping ability to handle disasters [5,6]. In a previous study, 98.4% of disaster response personnel rated the appropriateness of disaster triage as average or below, and 52.4% emphasized the need for specialized training in disaster triage methods [7]. Similar issues have been identified worldwide. Prior research indicates that many nurses demonstrate insufficient knowledge and confidence in disaster response, underscoring the need for practical, immersive training methods [8–10].

The role of nurses at disaster sites is emphasized, and disaster education is being developed for nursing students as future healthcare professionals [4,11]. According to expert Delphi surveys, accurate and rapid disaster triage is the most crucial topic in nursing education, highlighting the need to provide opportunities for experiential learning on efficient disaster triage [4,11]. Currently, disaster triage training mainly includes lectures on mass casualties, case-based learning, and tabletop exercises, with large-scale simulations using simulators and standardized patients also being implemented [12]. However, lectures, case-based learning, and tabletop exercises may be limited in their ability to help nursing students master disaster triage because they do not fully account for real-time changes in patient condition that can occur during disasters [11,12]. Moreover, although large-scale simulations can provide learning environments that effectively replicate disaster scenarios, they have drawbacks, such as requiring significant human and physical resources and providing limited continuous learning opportunities [12].

To address these limitations, the development of simulation training utilizing virtual reality (VR) has been expanding both domestically and internationally. VR-based simulation education is an effective method that realistically reproduces high-risk situations or environments that are otherwise difficult to replicate, allowing learners to acquire knowledge and skills through repeated experiential learning and improve their ability to make appropriate decisions [11,13]. In disaster situations characterized by uncertainty and mass casualties, healthcare professionals must use analytical cognitive processes quickly to perform triage based on medical knowledge [14]. Moreover, VR simulations can be repeatedly deployed across various institutions and settings with minimal physical and human resources, making them cost-effective. Thus, VR-based training provides a sustainable alternative to traditional methods for disaster preparedness education [14,15]. Furthermore, VR enables standardized training to be delivered even in resource-limited or geographically dispersed environments, thereby supporting educational equity by providing a consistent simulation experience regardless of institutional infrastructure [16,17]. Therefore, VR-based disaster triage simulation training is an effective method to support immersive, repeated, and equitable experiential learning in diverse educational settings.

In the unpredictable environment of a disaster site, nursing students must not only perform triage quickly and accurately but also develop confidence and cognitive flexibility by analyzing various casualty situations and making decisions accordingly [3,4,18,19]. Hammond’s theory of the cognitive continuum, indicates that simulation training mimicking real situations and comprising realistic and immersive tasks enables safe repetitive learning, thereby allowing learners to optimize their cognitive judgments based on information provided during the task [19]. This study aimed to develop and apply a VR-based disaster simulation training program to evaluate its effects on nursing students’ disaster triage accuracy, disaster triage time required, disaster triage confidence, learning immersion, and cognitive flexibility. Thus, this study is intended to propose a new direction for educational curricula that enhances the capabilities of nursing students, who will be tasked with performing effective triage following disasters in the future.

### Theoretical framework

At disaster sites with mass casualties, healthcare professionals must apply medical and disaster-related knowledge to assess the condition of those affected and perform efficient triage quickly and accurately. Cognitive continuum theory (CCT) posits that cognition moves along a continuum from intuition to analytical thinking, and individuals can execute efficient cognitive processes depending on the characteristics of a given task [20]. CCT suggests that intuition can facilitate fast and effective responses in complex decision-making environments where time or information is limited, but emphasizes the need for analytical processes when detailed and careful decision-making is possible [20]. In this context, disaster triage decision-making under uncertain conditions can be effectively addressed through simulation training that incorporates learning elements based on CCT [19].

Simulation theory underscores the importance of systematically applying learning tasks through the following stages: task goal preparation, pre-briefing, structured simulation execution, and reflection [21]. In this study, these stages were integrated into simulation learning elements based on CCT into VR-based disaster triage training. Supporting this approach, Pedersen et al. [19] explained the importance of analytical cognitive activities in nursing based on learners’ situational awareness as a factor that influences successful learning during simulation training. Cognitive learning elements include analytical cognition in tasks, situational awareness, cognitive control, and self-awareness elements [19]. The analytical cognition phase was structured to promote repeated case-based decision-making. During the VR simulation, learners repeatedly encountered six casualty cases, each presenting with diverse symptoms and clinical conditions. They were provided with limited time to apply the Simple Treatment and Rapid Transport (START) or Jump START triage protocols using observable clinical cues, such as respiratory rate, perfusion, and consciousness level. The task demanded rapid analysis and application of medical knowledge, thereby strengthening learners’ ability to make structured evidence-based decisions. The situational awareness phase was designed as a training component that utilized realistic sensory stimuli in a VR setting. Realistic audiovisual cues, such as sirens, crowd noise, smoke, visible injuries, and patient gestures, were integrated to simulate the complexity and urgency of actual mass casualty situations. The use of these stimuli aimed to foster context-sensitive clinical judgment and improve learners’ ability to process environmental cues effectively. The cognitive control phase focused on developing learners’

self-regulatory capacity in high-pressure scenarios. Learners were prompted to triage each case without external guidance, requiring them to manage time pressure and environmental distractions independently. The simulation was deliberately designed to be self-paced, and corrective feedback was withheld until all cases were complete. This approach was intended to support the internalization of decision-making strategies and reinforce cognitive discipline. The self-awareness phase was structured based on guided reflection questions. In the reflection stage, learners completed a debriefing protocol that included structured questions such as “What cues did you use to make your decision,” “What challenges did you encounter during triage,” and “How would you handle a similar situation differently?” This reflective process aims to facilitate self-assessment, promote deeper insight into learners’ clinical reasoning, and support the development of critical thinking and adaptive learning. This process was intended to improve the accuracy and speed of disaster triage, boost learners’ confidence in efficient decision-making, enhance learning immersion, and increase cognitive flexibility, thereby laying the groundwork for empirical learning processes in the disaster triage context.

## Materials and methods

### Study design

This study used a non-equivalent control group pretest-posttest design to evaluate the effectiveness of VR-based disaster simulation education for nursing students.

### Participants and sample size

Participants were selected using convenience sampling from third- and fourth-year nursing students at two universities in two cities with similar curricula. Recruitment notices were posted to ensure that students from both universities were included in the experimental and control groups to minimize bias and enhance the validity of the research design. The number of participants required was calculated using G*Power 3.1. Based on previous studies [22], the minimum sample size was determined using an independent t-test with a significance level of.05, a power of.80, and an effect size. The results indicated the need for 60 total participants, with 30 per group. Considering potential dropouts, an additional 20% were recruited, resulting in 36 participants per group.

Students met the eligibility criteria if they had completed at least two semesters of basic nursing practice, understood the purpose of the study, and provided written consent for voluntary participation. Those who had previously participated in disaster simulation training or programs or had experienced side effects, such as motion sickness, from VR programs were excluded. Among the initially recruited participants, five were excluded, including three who provided surveys with missing responses and two who withdrew for personal reasons. The final analysis included 33 participants in the experimental group and 34 in the control group.

### Data collection

Data were collected after receiving IRB approval from K University (KNU2023−0010). The collection period spanned from January 11 to April 30, 2023. After recruitment announcements were made at two universities located in D and K cities, recruitment notices were posted for the experimental and control groups, and those interested were selected on a first-come, first-served basis. The notices were posted on the websites and social media of both universities, and explanations and consent forms were provided to participants recruited via telephone calls and QR codes. The researcher reviewed the selection and exclusion criteria, and the participants gathered at the designated locations for each school and signed consent forms. The experimental and control groups signed consent forms that included the purpose and content of the VR-based and written simulations, respectively. Both consent forms included details regarding the protection of the participants’ personal data during data collection and possibility of withdrawing from the study at any time without punishment and explained relevant matters. The program dates and data collection periods were kept separate to minimize cross-contamination between the experimental and control groups. Participants received a small gift at the end of the study and those in the control group received the VR program after the end of the study.

### Virtual reality-based disaster simulation education

#### Disaster simulation development.

**Fig 1:**
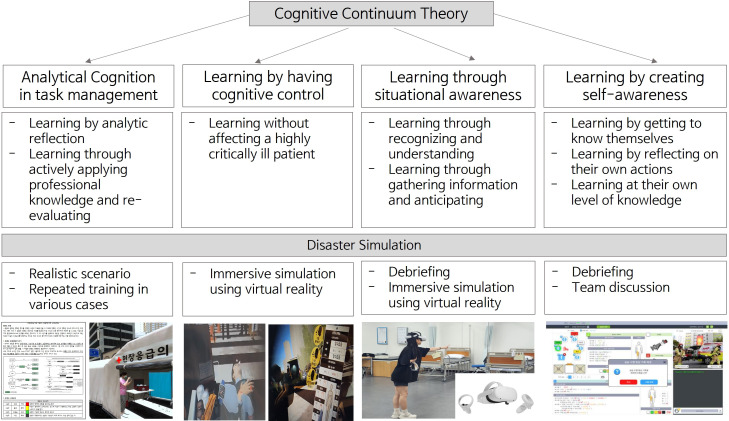
Design of the virtual reality disaster simulation education program based on cognitive continuum theory.

The simulation incorporated analytical cognition in task management, learning through situational awareness, learning by having cognitive control, and learning by creating self-awareness [19] (Fig 1). The analytical cognitive learning elements included learning through analytical reflection and actively applying specialized knowledge [19]. The knowledge provided during pre-learning and obtained through simulations and specific realistic scenarios was structured to facilitate easier application.. The VR environment was designed to increase immersion, aiming to promote analytical thinking and active knowledge application. Repeated training sessions were embedded to reinforce key triage principles and allow for repeated knowledge application across varied scenarios. Cognitive control learning elements were included to allow learners to engage in clinical reasoning in a safe environment without risk to actual patients. The simulation was structured to guide learner engagement with both the technical and non-technical aspects of disaster care. Learning through situational awareness was enabled, which allowed learners to recognize and understand situations that encompass learning and develop new perspectives and engage in self-reflection through debriefing. Team activities were structured to support information acquisition and organization, aiming to enhance collaborative learning experiences. Finally, the debriefing phase was designed to encourage learners to reflect on their actions and explore their learning processes to gain a deeper understanding. Specifically, a structured debriefing component was included to encourage learners to reflect on their decisions, identify knowledge gaps, and formulate strategies for future actions.

The START triage method was used to classify patient severity. This method checks the ability to walk at level 1, spontaneous breathing at level 2, respiratory rate at level 3, perfusion at level 4, and mental status at level 5 and distinguishes between minor, delayed, immediate, and expectant. This program is designed to classify severity according to age by applying both START triage for adults and Jump START triage for children. In a VR situation, the patient’s breathing rate and pulse were measured directly, and their level of consciousness was confirmed by asking their name or about the weather. For realistic expression, all patient injuries were expressed to enable emergency treatment, such as applying a tourniquet or ambu-bag. The software provided to the experimental group was deemed appropriate, with a content validity index (CVI) of.80 or above, as assessed by two nursing professors, one disaster educator, and one nurse. The simulation involved performing triage for six patients in a disaster scenario.

### Application of disaster simulation

**Fig 2:**
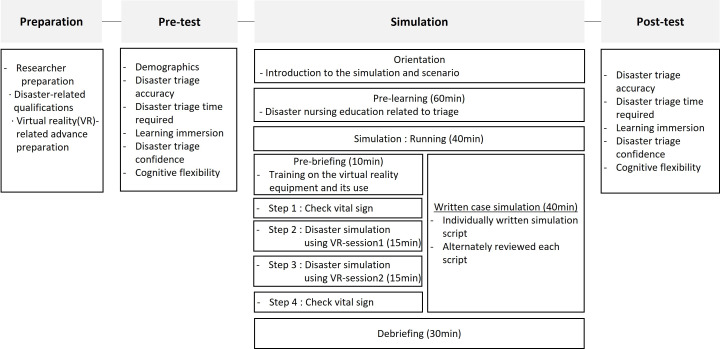
Study design.

This study was conducted in four stages (Fig 2). The first stage was the researcher preparation stage, in which two instructors had experience operating VR simulations, BDLS (Basic Disaster Life Support) instructor qualifications, and disaster nursing education. A VR-based disaster simulation was conducted for approximately 30 minutes before the study to maintain consistency in the intervention time and methods for both the experimental and control groups, ensuring reliability.

The second stage was the pretest stage, which was conducted in a classroom setting. To prevent experimental contamination, the study was performed with the experimental group in the morning and the control group in the afternoon at separate locations, with data collected in seminar rooms approved by each university.

The third stage was the simulation stage, which lasted approximately 2 hours and 10 minutes. Both groups received nursing education related to disaster triage for 60 minutes as pre-learning. The experimental group then individually completed health self-assessment checklists and consent forms. The experimental group received training on the VR equipment and its use during a 10-minute pre-briefing in a 5m x 9m VR practice room at their school. The 30-minute VR-based disaster simulation training was divided into two sessions of approximately 15 minutes each to reduce VR side effects. The equipment used in the VR simulation was Oculus Quest 2, and a guardian system was implemented to ensure participants’ safety by turning off the virtual screen if they left the set space. Before the VR-based disaster simulation training, the experimental group’s device information and condition, space utilization, and hygiene inspection, and the users’ health and cognitive status were checked, and changes in condition were monitored during and after use to ensure safety. The control group received written disaster scenario-based education in a classroom for 40 minutes. The session was facilitated by an experienced instructor with a background in disaster nursing and used printed materials that presented the same scenarios as those in the VR simulation. Participants reviewed severity classification cases and practiced decision-making through guided worksheets and small-group discussions. The educational content was designed to match the learning objectives of the experimental group’s VR experience, excluding the immersion component. This ensured content consistency while preserving the methodological distinction between interventions.

Both groups underwent a 30-minute debriefing followed by a post-survey before the study was concluded. Participants were debriefed individually, and the experimental and control groups were provided with time to reflect on and learn from the knowledge included in the program. Independent research assistants administered the surveys to ensure methodological transparency and minimize bias. However, the evaluators were not blinded to the participants’ group assignments..

## Measurement

### Disaster triage accuracy

Disaster triage accuracy was assessed using the disaster mass triage tool developed by Park and Hwang [23], based on the SALT (Sort, Assess, Life-saving intervention and Treatments and/or transport) multiple injury severity classification system and START triage method, as outlined in South Korea’s Emergency Medical Response Manual for Disasters. Park and Hwang [23] developed their five-item measurement tool based on scenarios. Answers are scored using a dichotomous scale, with correct answers receiving 1 point and incorrect answers receiving 0 points. Total scores range from 0 to 5, with higher scores indicating better disaster triage accuracy. The scenarios used for triage training in the National Disaster Life Support professional courses (American Medical Association [AMA], 2012) and those by Park and Choi (2012) [24] were referenced. This measurement tool, as revised by Park and Hwang [24], was validated by three nursing college professors, with a CVI of 0.96.

### Disaster triage time required

The time required for disaster triage was measured using Park and Hwang’s [23] tool. The researcher recorded in seconds the time it took each participant to select their responses to the disaster triages score tool. In a previous study, the average time taken to classify a single casualty was 15.6 seconds [24].

### Learning immersion

Yu’s [25] learning immersion tool uses a modified measure based on that of Engeser and Rheinberg [26]. The tool comprises 10 items rated on a 5-point Likert scale and measures the learner’s immersive experience during simulation. Learning immersion is the optimal psychological state in which learners are immersed and focused when performing a task. Thus, for the modified scale, higher scores indicate greater concentration during simulation practice [25]. Cronbach’s alpha was.84 in Yu [25] and.71 in this study.

### Disaster triage confidence

Participants’ self-perceived confidence in disaster triage was assessed using a tool adapted by Park and Hwang (2024) [24], referencing the Korean Nursing Education Evaluation Institute’s standard guidelines for simulation practices. The tool comprises one question that asks participants to rate their current self-confidence in disaster nursing on a scale ranging from 0 (not confident) to 10 (very confident). Higher scores indicate greater self-confidence in disaster nursing.

### Cognitive flexibility

This study used the Cognitive Flexibility Inventory (CFI) developed by Dennis and Vander Wal (2010) [27] and adapted and validated by Hur (2011) [28]. The CFI comprises 19 items: 11 on the alternative subscale, which measures the ability to conceive of various alternative explanations for life events and behaviors, and 8 on the control subscale, which assesses perceived control in difficult situations. Responses are rated on a 5-point Likert scale, with higher scores indicating greater cognitive flexibility. Cronbach’s alpha was.86 in Hur [28] and.72 in this study.

### Data analysis

The collected data were analyzed using SPSS 27.0. Participants’ general characteristics were analyzed using frequencies, percentages, means, and standard deviations. Homogeneity was assessed between the experimental and control groups using chi-square and independent t-tests. Independent t-tests were used to verify the effects between the experimental and control groups. The reliability of the research tools was calculated using Cronbach’s alpha.

## Results

### Homogeneity test of general characteristics and variables between groups

Table 1 presents the participants’ general characteristics and homogeneity of the variables. The experimental group was 18.2% male and 81.8% female, and the control group was 11.8% male and 88.2% female, with no statistically significant difference between groups (**p* *= .461). Mean age (**p* *= .950), academic performance (**p* *= .721), satisfaction with major (**p* *= .500), satisfaction with clinical practice (**p* *= .918), simulation experience (**p* *= .297), VR experience (**p* *= .217), and disaster experience (**p* *= .151) were homogeneous between the experimental and control groups. Pre-study variables, such as disaster triage accuracy (t = 0.46, **p* *= .645), disaster triage time required (t = −0.18, **p* *= .855), disaster triage confidence (t = 1.11, **p* *= .271), learning immersion (t = −1.65, **p* *= .104), and cognitive flexibility (t = −0.33, **p* *= .745), were also homogeneous between groups (Table 1).

**Table 1:** General Participant Characteristics (N = 67).

Characteristics	Categories	Exp. (n = 33)	Cont. (n = 34)	χ^2^ or t	*P*
		n(%) or M ± SD	n(%) or M ± SD		
Gender	Male	6 (18.2)	4 (11.8)	0.54	.461
	Female	27 (81.8)	30 (88.2)		
Age (yrs)		21.45 ± 1.00	21.47 ± 1.08	−0.06	.950
Academic performance (CAP)	> 4.0	12 (36.4)	8 (23.5)	1.34	.721
	3.5–4.0	7 (21.2)	9 (26.5)		
	3.0–3.5	8 (24.2)	10 (29.4)		
	< 3.0	6 (18.2)	7 (20.6)		
Satisfaction with major	Satisfied	22 (66.7)	19 (55.9)	1.39	.500
	Moderate	10 (30.3)	12 (35.3)		
	Dissatisfied	1 (3.0)	3 (8.8)		
Satisfaction with clinical practice	Satisfied	14 (42.4)	14 (41.2)	0.01	.918
	Moderate	19 (57.6)	20 (58.8)		
Simulation experience	Yes	26 (78.8)	30 (88.2)	1.09	.297
	No	7 (21.2)	4 (11.8)		
VR experience	Yes	8 (24.2)	13 (38.2)	1.52	.217
	No	25 (75.8)	21 (61.8)		
Disaster experience	Yes	9 (27.3)	15 (44.1)	2.07	.151
	No	24 (72.7)	19 (55.9)		
Disaster triage accuracy		2.33 ± 0.60	2.26 ± 0.62	0.46	.645
Disaster triage time required		91.52 ± 9.98	91.91 ± 7.64	−0.18	.855
Disaster triage confidence		5.52 ± 1.25	5.18 ± 1.24	1.11	.271
Learning immersion		3.51 ± 0.33	3.63 ± 0.26	−1.65	.104
Cognitive flexibility		3.51 ± 0.29	3.53 ± 0.31	−0.33	.745

Table notes Exp. = Experimental group; Cont. = Control group; CAP = Cumulative Average Point; VR = Virtual Reality.

### Effectiveness of disaster simulation education based on virtual reality

Table 2 presents the results on the effectiveness of VR-based disaster triage simulation training. Disaster triage accuracy improved between the pretest and posttest from 2.33 ± 0.60 to 4.03 ± 0.47 in the experimental group and from 2.26 ± 0.62 to 2.94 ± 0.34 in the control group. The post-pre difference was 1.70 ± 0.81 in the experimental group and 0.68 ± 0.54 in the control group, with a statistically significant difference between groups (t = 6.11, **p* *< .001). Disaster triage time decreased from 91.52 ± 9.98 to 72.61 ± 5.33 seconds in the experimental group and from 91.91 ± 7.64 to 77.560 ± 5.57 seconds in the control group. The post-pre difference was −18.97 ± 9.99 in the experimental group and −14.53 ± 8.72 in the control group; however, this difference was not statistically significant between groups (t = −1.94, **p* *= .057). Disaster triage confidence increased from 5.52 ± 1.25 to 7.48 ± 1.18 in the experimental group and from 5.18 ± 1.24 to 5.94 ± 0.95 in the control group. The post-pre difference was 1.97 ± 1.94 in the experimental group 0.76 ± 1.72 in the control group, with a statistically significant difference between groups (t = 2.69, **p* *= .009). Learning immersion increased from 3.51 ± 0.33 to 4.35 ± 0.29 in the experimental group and from 3.63 ± 0.26 to 3.85 ± 0.35 in the control group. The post-pre difference was 0.88 ± 0.42 in the experimental group and 0.17 ± 0.43 in the control group, with a statistically significant difference between two groups (t = 6.88, **p* *< .001). Cognitive flexibility increased from 3.51 ± 0.33 to 3.79 ± 0.23 in the experimental group, but decreased from 3.53 ± 0.31 to 3.34 ± 0.25 in the control group. The post-pre difference was 0.28 ± 0.36 in the experimental group and −0.20 ± 0.35 in the control group, with a statistically significant difference between groups (t = 5.47, *p* < .001).

**Table 2:** Effectiveness of virtual reality-based disaster simulation education (N = 67).

Variables	Pretest		Posttest		Difference		t	*p*
	Exp. (n = 33)	Cont. (n = 34)	Exp. (n = 33)	Cont. (n = 34)		Exp. (n = 33)	Cont. (n = 34)
Disaster triage accuracy	2.33 ± 0.60	2.26 ± 0.62	4.03 ± 0.47	2.94 ± 0.34	1.70 ± 0.81	0.68 ± 0.54	6.11	<.001
Disaster triage time required	91.52 ± 9.98	91.91 ± 7.64	72.61 ± 5.33	77.56 ± 5.57	−18.97 ± 9.99	−14.53 ± 8.72	−1.94	.057
Disaster triage confidence	5.52 ± 1.25	5.18 ± 1.24	7.48 ± 1.18	5.94 ± 0.95	1.97 ± 1.94	0.76 ± 1.72	2.69	.009
Learning immersion	3.51 ± 0.33	3.63 ± 0.26	4.35 ± 0.29	3.85 ± 0.35	0.88 ± 0.42	0.17 ± 0.43	6.88	<.001
Cognitive flexibility	3.51 ± 0.29	3.53 ± 0.31	3.79 ± 0.23	3.34 ± 0.25	0.28 ± 0.36	−0.20 ± 0.35	5.47	<.001

Table notes Exp. = Experimental Group; Cont. = Control Group; VR = Virtual Reality.

## Discussion

This study aimed to develop a VR-based disaster triage simulation and validate its effectiveness among nursing students, thereby demonstrating the utility of a new educational curriculum. The experimental group, who participated in the VR-based disaster triage simulation, showed significant improvement in disaster triage accuracy compared to the control group, who used a written case simulation. Although direct comparison is difficult because of the use of different measurement tools, previous studies that have utilized mobile virtual practice [29] or applied VR-based programs [30] for triage education have also reported improved triage accuracy, supporting the results of this study. Prior research has shown that highly realistic simulation scenarios enhance learners’ analytical thinking [19]. Problem-based learning, high-fidelity simulator training, and web-based simulation education have shown limitations in creating realistic situations when used in safety and disaster nursing, whereas VR-based simulation is an educational method that compensates for these limitations [31]. Therefore, the realistic and specific cases applied in this study’s VR simulation likely improved disaster triage accuracy by enabling learners to actively utilize their knowledge and learn analytical cognitive processes.

The disaster triage time required decreased in both the experimental and control groups after they completed their respective programs. However, no significant difference was found between groups. These findings may stem from the designs of the VR and written simulations, which repeatedly exposed learners to realistic patient scenarios in complex disaster contexts. This allowed learners to engage continuously in repeated training. Thus, both groups required less time for disaster triage following the intervention. These designs likely helped improve the triage speeding both groups. Previous studies have reported that written disaster triage took approximately 15.6 seconds per case for nurses [24] and approximately 18.7 seconds for nursing students [32], whereas this study reported 18.3 seconds. However, a prior study in which nursing students engaged in a mobile-based disaster triage game in the experimental group and traditional disaster triage education in the control group also showed decreased classification time post-intervention without significant differences between groups [32], which supports the results of this study. These findings align with those of the present study and suggest that both interventions can effectively improve performance. Notably, both the prior study and this study implemented approximately 30 minutes of triage simulation training, which may explain the lack of a significant difference between groups. Meaningful differences in triage time may be difficult to observe after a single-session intervention [32]. When comparing the decision-making response time for Marines in assessing tactical situations and formulating battle plans, no statistically significant difference was found between highly experienced and less experienced groups under conditions of low uncertainty. However, under high-uncertainty conditions, the highly experienced group demonstrated significantly shorter execution times for their chosen course of action [33]. These findings suggest that while decision response time can be improved through training, differences in response efficiency may still emerge depending on the complexity of the situation and the responder’s level of experience. Nevertheless, the VR-based simulation training in this study reduced the average triage time to 14.5 seconds, which is faster than the 15 seconds reported in a prior study using a smartphone-based game [32] and the 15.6 seconds reported in a study involving nurses with disaster triage experience [24]. These findings suggest that repetitive disaster triage learning in an immersive VR setting can effectively reduce the time nursing students require for triage. However, nursing students must consider both speed and accuracy in disaster triage to enhance the prognosis of multiple casualties; thus, further studies using measurement tools that assess both are required. Additionally to reduce triage time in high-uncertainty disaster situations, training should incorporate repetitive exposure to diverse scenarios, enabling individuals to develop adaptive decision-making skills under complex and unpredictable conditions.

Disaster triage confidence significantly improved in the experimental group compared to the control group. This aligns with prior studies showing that repeated learning in a realistic VR environment enhances nursing performance confidence [34,35]. Additionally, studies have shown that VR-based disaster response education is more effective than traditional low-fidelity simulator training and can increase learners’ performance confidence [30], supporting this study’s findings. The VR-based simulation in this study likely improved learners’ disaster triage confidence by providing a safe environment in which they could gain mastery experience without harming patients. However, the disaster triage confidence measure used in this study relied on self-reported data, which are inherently subjective and may be influenced by individual perceptions or response bias. Future research should be strengthened by using objective performance-based metrics, such as behavioral observations, biomarkers, or standardized assessment tools, to complement subjective confidence measures and provide a more comprehensive understanding of training effects.

Learning immersion was significantly enhanced in the experimental group compared to the control group. VR simulations, which stimulate all senses in a 3D interactive mode rather than only delivering visual data unilaterally, maximize user immersion [35,36], which aligns with this study’s findings. Learning immersion, which is the state of being engrossed in an educational task, not only provides learners with satisfaction from the immersive experience itself but also enhances intrinsic motivation [37], creating a foundation for learners to actively integrate skills and knowledge. Thus, future studies should qualitatively analyze the elements that enhance VR immersion to identify and apply these components.

Cognitive flexibility was significantly enhanced in the experimental group compared to the control group. Cognitive flexibility is the ability to reconfigure judgments effectively in rapidly changing situations [27,28] and is crucial for making timely decisions in disaster triage. The high-fidelity VR simulation developed in this study could induce analytical and strategic cognitive processes, even in situations that might otherwise focus on intuitive cognitive processes [19]. The process of prioritizing and reflecting on various cases developed in this study [38] provides a higher-level learning experience that contributes to learners’ ability to respond effectively to rapidly changing disaster situations, thereby enhancing cognitive flexibility.

Therefore, the VR-based simulation education developed in this study not only increased knowledge levels but also improved accuracy, confidence, learning immersion, and cognitive flexibility in disaster triage. This underscores the significance of the application and potential expansion of future-oriented methods in VR-based disaster triage simulation education.

## Conclusion

This study is academically significant because it develops and validates a VR-based disaster triage simulation training for nursing students, who are potential first responders at disaster sites, thus laying the groundwork for a future-oriented disaster nursing education curriculum. Moreover, the training developed in this study was found to enhance nursing students’ accuracy, confidence, learning immersion, and cognitive flexibility in disaster triage. This has clinical significance because these skills can help nurses respond effectively to highly uncertain disaster scenes and potentially reduce mortality and morbidity rates among multiple casualties.

This study had some limitations. First, it used a convenience sample of nursing students from two universities, which limits the generalizability of the results. Second, it was not possible to measure the long-term effects of the applied program or apply a tool that integrates disaster triage speed and accuracy, suggesting the need for further replication studies. Third, this study could not determine the sustainability of the effects of VR simulation. Therefore, future studies should include a follow-up test after the posttest to evaluate long-term learning effects. Fourth, this study verified the effectiveness of the intervention only among nurses and not among other healthcare professionals. Therefore, future studies are required to verify the effectiveness of the developed intervention in various healthcare-related occupations, such as emergency medical technicians and medical students.

## Supporting information

S1 Table General characteristics dataset of participants. (DOCX)

S2 Table General characteristics dataset of participants. (DOCX)

S3 File Video about simulation learning. (MP4)

## References

[1] Heldring S, Jirwe M, Wihlborg J, Berg L, Lindström V. Using High-Fidelity Virtual Reality for Mass-Casualty Incident Training by First Responders - A Systematic Review of the Literature. Prehosp Disaster Med. 2024;39(1):94–105. doi: 10.1017/S1049023X24000049 38328887 PMC10882557

[2] Elmhadhbi L, Karray M-H, Archimède B, Otte JN, Smith B. Ontology-Driven Multicriteria Decision Support for Victim Evacuation. Int J Info Tech Dec Mak. 2021;21(01):243–72. doi: 10.1142/s021962202150053x

[3] Goniewicz K, Goniewicz M, Burkle FM, Khorram-Manesh A. Cohort research analysis of disaster experience, preparedness, and competency-based training among nurses. PLoS One. 2021;16(1):e0244488. doi: 10.1371/journal.pone.0244488 33417601 PMC7793243

[4] Kim C, Han SY, Chin YR. A Delphi Study for Development of Disaster Nursing Education Contents in Community Health Nursing. J Korean Acad Community Health Nurs. 2021;32(4):555. doi: 10.12799/jkachn.2021.32.4.555

[5] Kim MY, Kim MS. Correlation among nurses’ educational status, knowledge and disaster preparedness abilities. J Korea Academia-Ind Coop Soc. 2017;18(7):589–98. doi: 10.5762/KAIS.2017.18.7.589

[6] Kalanlar B. Effects of disaster nursing education on nursing students’ knowledge and preparedness for disasters. International Journal of Disaster Risk Reduction. 2018;28:475–80. doi: 10.1016/j.ijdrr.2017.12.008

[7] Yoon JH. The importance of recognition and professional training for triage in disasters and mass casualty incidents. M.Sc. Thesis, Kyungpook National University. 2014. https://www.riss.kr/link?id=T13554446

[8] Alim S, Kawabata M, Nakazawa M. Evaluation of disaster preparedness training and disaster drill for nursing students. Nurse Educ Today. 2015;35(1):25–31. doi: 10.1016/j.nedt.2014.04.016 24832532

[9] Veenema TG, Griffin A, Gable AR, MacIntyre L, Simons RN, Couig MP, et al. Nurses as Leaders in Disaster Preparedness and Response--A Call to Action. J Nurs Scholarsh. 2016;48(2):187–200. doi: 10.1111/jnu.12198 26869230

[10] Jennings-Sanders A, Frisch N, Wing S. Nursing students’ perceptions about disaster nursing. Disaster Manag Response. 2005;3(3):80–5. doi: 10.1016/j.dmr.2005.04.001 15986028

[11] Park SK, Kim HJ. Development and Evaluation of Virtual Reality-based Simulation Content for Nursing Students Regarding Emergency Triage. J Korean Acad Fundam Nurs. 2023;30(2):292–301. doi: 10.7739/jkafn.2022.30.2.292

[12] Kman NE, Price A, Berezina-Blackburn V, Patterson J, Maicher K, Way DP. First Responder Virtual Reality Simulator to Train and Assess Emergency Personnel for Mass. Journal of Emergency Management. 2023;12(3):123–30. doi: 10.1002/emp2.12903 36817080 PMC9933861

[13] Verkuyl M, Hughes M. Virtual Gaming Simulation in Nursing Education: A Mixed-Methods Study. Clin Simul Nurs. 2019;29:9–14 doi: 10.1016/j.ecns.2019.02.001

[14] Hamdi A, Al Thobaity A. Enhancing Disaster Triage Competencies through Simulation-Based Training: An Interventional Study among Undergraduate Nursing Students. Sustainability. 2023;15(21):15513. doi: 10.3390/su152115513

[15] Alshowair A, Bail J, AlSuwailem F, Mostafa A, Abdel-Azeem A. Use of virtual reality exercises in disaster preparedness training: A scoping review. SAGE Open Med. 2024;12:20503121241241936. doi: 10.1177/20503121241241936 38623475 PMC11017811

[16] Hsu EB, Li Y, Bayram JD, Levinson D, Yang S, Monahan C. State of virtual reality based disaster preparedness and response training. PLoS Curr. 2013;5:ecurrents.dis.1ea2b2e71237d5337fa53982a38b2aff. doi: 10.1371/currents.dis.1ea2b2e71237d5337fa53982a38b2aff 23653102 PMC3644293

[17] Please H, Narang K, Bolton W, Nsubuga M, Luweesi H, Richards NB, et al. Virtual reality technology for surgical learning: qualitative outcomes of the first virtual reality training course for emergency and essential surgery delivered by a UK-Uganda partnership. BMJ Open Qual. 2024;13(1):e002477. doi: 10.1136/bmjoq-2023-002477 38286564 PMC10826552

[18] Feng X, Perceval GJ, Feng W, Feng C. High Cognitive Flexibility Learners Perform Better in Probabilistic Rule Learning. Front Psychol. 2020;11:415. doi: 10.3389/fpsyg.2020.00415 32231624 PMC7083211

[19] Pedersen I, Lee Solevåg A, Solberg MT. Simulation-Based Training Promotes Higher Levels of Cognitive Control in Acute and Unforeseen Situations. Clinical Simulation in Nursing. 2019;34:6–15. doi: 10.1016/j.ecns.2019.05.003

[20] Cader R, Campbell S, Watson D. Cognitive Continuum Theory in nursing decision-making. J Adv Nurs. 2005;49(4):397–405. doi: 10.1111/j.1365-2648.2004.03303.x 15701154

[21] Watts PI, McDermott DS, Alinier G, Charnetski M, Ludlow J, Horsley E, et al. Healthcare Simulation Standards of Best Practice: Simulation Design. Clin Simul Nurs. 2021;58:14–21. doi: 10.1016/j.ecns.2021.09.004

[22] Yoo M, Kim J, Koo Y, Song J. A meta-analysis on effects of VR, AR, MR-based learning in Korea. KAFEIAM. 2018;24(3):459–88. doi: 10.15833/kafeiam.24.3.459

[23] Park YM, Hwang WJ. Development and Effect of a Simulation-Based Disaster Nursing Education Program for Nursing Students Using Standardized Patients. J Nurs Res. 2024;32(1):e314. doi: 10.1097/jnr.0000000000000596 38265077

[24] Park JY, Choi SM. A study on the triage performance of military nurses and its related factors using a mass casualty scenario, paper exercise. Korean Journal of Military Nursing Research. 2012;30:128–42.

[25] Yoo J-H, Kim Y-J. Factors Influencing Nursing Students’ Flow Experience during Simulation-Based Learning. Clinical Simulation in Nursing. 2018;24:1–8. doi: 10.1016/j.ecns.2018.09.001

[26] Engeser S, Rheinberg F. Flow, performance and moderators of challenge-skill balance. Motiv Emot. 2008;32(3):158–72. doi: 10.1007/s11031-008-9102-4

[27] Dennis JP, Vander Wal JS. The Cognitive Flexibility Inventory: Instrument Development and Estimates of Reliability and Validity. Cogn Ther Res. 2009;34(3):241–53. doi: 10.1007/s10608-009-9276-4

[28] Heo S. The Role of Cognitive Flexibility in the Relationship between Perfectionism and Psychological Maladjustment. Doctoral dissertation, Seoul National University; 2011. https://www.riss.kr/link?id=T12383789

[29] Park J-S, Shin H-H. Verification of the Effectiveness of Education and Training by Triage of Multiple Casualties Using Mobile Virtual Practice. Fire Sci Eng. 2021;35(5):74–80. doi: 10.7731/kifse.739137da

[30] Thompson S. Mass casualty triage: using virtual reality in hazardous area response teams training. Journal of Paramedic Practice. 2023;15(10):418–27. doi: 10.12968/jpar.2023.15.10.418

[31] Kim Y, Kim WJ, Min H young. Nursing students’ experiences in virtual simulation practice. J Korean Acad Soc Nurs Educ. 2020;26(2):198–207. doi: 10.5977/jkasne.2020.26.2.198

[32] Lee J. The effects of smartphone based serious educational game for disaster casualty triage in nursing students. D. Thesis, Kyungpook National University. 2018. https://www.riss.kr/link?id=T14745164

[33] Kobus DA, Proctor S, Holste S. Effects of experience and uncertainty during dynamic decision making. International Journal of Industrial Ergonomics. 2001;28(5):275–90. doi: 10.1016/s0169-8141(01)00022-1

[34] Ahn MK, Lee CM. Development and Effects of Head-Mounted Display-Based Home-Visits Virtual Reality Simulation Program for Nursing Students. J Korean Acad Nurs. 2021;51(4):465–77. doi: 10.4040/jkan.21051 34497255

[35] Plotzky C, Loessl B, Kuhnert B, Friedrich N, Kugler C, König P, et al. My hands are running away - learning a complex nursing skill via virtual reality simulation: a randomised mixed methods study. BMC Nurs. 2023;22(1):222. doi: 10.1186/s12912-023-01384-9 37370124 PMC10294322

[36] Lee E, Baek G, Hwang Y. Effectiveness of the Patient’s Severity Classification Competency Promotion Virtual Reality Program of Nursing Students during the COVID-19 Pandemic Period. Healthcare (Basel). 2023;11(8):1122. doi: 10.3390/healthcare11081122 37107957 PMC10137825

[37] Steele JP, Fullagar CJ. Facilitators and outcomes of student engagement in a college setting. J Psychol. 2009;143(1):5–27. doi: 10.3200/JRLP.143.1.5-27 19157070

[38] Rivera J. Cognitive flexibility: using mental simulation to improve script adaptation. M.Sc. Thesis, Thesis, University of Central Florida. 2016. https://stars.library.ucf.edu/etd/5186

