# Supplementary material for: Effects of virtual reality-based disaster simulation education on nursing students
Source: PLoS One. 2025 Oct 7;20(10):e0329563. doi: 10.1371/journal.pone.0329563 (PMC12503233; doi:10.1371/journal.pone.0329563)
Supplement: S1 Table — (DOCX) [file pone.0329563.s001.docx]

S1 Table. General characteristics dataset of participants

| Group | Gender | Age | Academic performance | Satisfaction  with major | Clinical practice  satisfaction | Simulation experience | VR experience | Disaster experience |
| --- | --- | --- | --- | --- | --- | --- | --- | --- |
| 2 | 2 | 21 | 2 | 1 | 1 | 1 | 1 | 1 |
| 2 | 2 | 20 | 1 | 1 | 1 | 2 | 2 | 2 |
| 2 | 2 | 21 | 3 | 1 | 2 | 1 | 2 | 2 |
| 2 | 2 | 20 | 4 | 2 | 2 | 1 | 2 | 2 |
| 2 | 2 | 21 | 2 | 2 | 2 | 1 | 2 | 2 |
| 2 | 2 | 20 | 2 | 2 | 2 | 1 | 1 | 1 |
| 2 | 2 | 22 | 3 | 1 | 1 | 1 | 2 | 2 |
| 2 | 2 | 21 | 1 | 1 | 1 | 1 | 1 | 1 |
| 2 | 1 | 24 | 4 | 3 | 2 | 1 | 1 | 1 |
| 2 | 2 | 21 | 3 | 1 | 1 | 1 | 1 | 1 |
| 2 | 2 | 20 | 1 | 1 | 1 | 1 | 2 | 2 |
| 2 | 2 | 20 | 1 | 1 | 2 | 1 | 2 | 2 |
| 2 | 2 | 21 | 1 | 1 | 1 | 2 | 2 | 2 |
| 2 | 2 | 23 | 3 | 1 | 2 | 1 | 2 | 2 |
| 2 | 1 | 22 | 4 | 2 | 2 | 1 | 2 | 2 |
| 2 | 1 | 21 | 2 | 2 | 2 | 1 | 2 | 2 |
| 2 | 1 | 21 | 2 | 2 | 2 | 1 | 1 | 1 |
| 2 | 2 | 21 | 3 | 1 | 1 | 1 | 2 | 2 |
| 2 | 2 | 22 | 1 | 1 | 1 | 1 | 1 | 1 |
| 2 | 2 | 22 | 4 | 3 | 2 | 1 | 1 | 1 |
| 2 | 2 | 22 | 3 | 1 | 1 | 1 | 1 | 1 |
| 2 | 2 | 23 | 1 | 1 | 1 | 2 | 2 | 2 |
| 2 | 2 | 22 | 3 | 1 | 2 | 1 | 2 | 2 |
| 2 | 2 | 23 | 4 | 2 | 2 | 1 | 2 | 2 |
| 2 | 2 | 22 | 2 | 2 | 2 | 1 | 2 | 2 |
| 2 | 2 | 22 | 2 | 2 | 2 | 1 | 1 | 1 |
| 2 | 2 | 21 | 3 | 1 | 1 | 1 | 2 | 2 |
| 2 | 2 | 21 | 1 | 1 | 1 | 2 | 2 | 2 |
| 2 | 2 | 21 | 3 | 1 | 2 | 1 | 2 | 2 |
| 2 | 2 | 22 | 4 | 2 | 2 | 1 | 2 | 2 |
| 2 | 2 | 22 | 2 | 2 | 2 | 1 | 2 | 2 |
| 2 | 2 | 24 | 2 | 2 | 2 | 1 | 1 | 1 |
| 2 | 2 | 21 | 4 | 3 | 2 | 1 | 1 | 1 |
| 2 | 2 | 20 | 3 | 1 | 1 | 1 | 1 | 1 |
| 1 | 2 | 20 | 1 | 1 | 1 | 2 | 2 | 2 |
| 1 | 2 | 21 | 3 | 1 | 2 | 1 | 2 | 2 |
| 1 | 1 | 21 | 4 | 2 | 2 | 1 | 2 | 2 |
| 1 | 1 | 20 | 2 | 2 | 2 | 1 | 2 | 2 |
| 1 | 1 | 21 | 2 | 2 | 2 | 1 | 1 | 1 |
| 1 | 1 | 20 | 1 | 1 | 1 | 2 | 2 | 2 |
| 1 | 2 | 22 | 1 | 1 | 1 | 1 | 1 | 1 |
| 1 | 2 | 21 | 4 | 3 | 2 | 1 | 1 | 1 |
| 1 | 2 | 20 | 3 | 1 | 1 | 1 | 1 | 1 |
| 1 | 2 | 20 | 1 | 1 | 1 | 1 | 2 | 2 |
| 1 | 1 | 21 | 1 | 1 | 2 | 1 | 2 | 2 |
| 1 | 2 | 23 | 1 | 1 | 1 | 2 | 2 | 2 |
| 1 | 1 | 22 | 3 | 1 | 2 | 1 | 2 | 2 |
| 1 | 2 | 21 | 4 | 2 | 2 | 1 | 2 | 2 |
| 1 | 2 | 21 | 2 | 1 | 1 | 1 | 1 | 1 |
| 1 | 2 | 21 | 1 | 1 | 1 | 2 | 2 | 2 |
| 1 | 2 | 21 | 3 | 1 | 2 | 1 | 2 | 2 |
| 1 | 2 | 21 | 4 | 2 | 2 | 1 | 2 | 2 |
| 1 | 2 | 22 | 2 | 2 | 2 | 1 | 2 | 2 |
| 1 | 2 | 22 | 2 | 2 | 2 | 1 | 1 | 1 |
| 1 | 2 | 24 | 1 | 1 | 1 | 1 | 2 | 2 |
| 1 | 2 | 21 | 1 | 1 | 2 | 1 | 2 | 2 |
| 1 | 2 | 22 | 1 | 1 | 1 | 2 | 2 | 2 |
| 1 | 2 | 22 | 3 | 1 | 2 | 1 | 2 | 2 |
| 1 | 2 | 22 | 4 | 2 | 2 | 1 | 2 | 2 |
| 1 | 2 | 22 | 2 | 1 | 1 | 1 | 1 | 1 |
| 1 | 2 | 22 | 1 | 1 | 1 | 2 | 2 | 2 |
| 1 | 2 | 24 | 3 | 1 | 2 | 1 | 2 | 2 |
| 1 | 2 | 22 | 2 | 2 | 2 | 1 | 1 | 1 |
| 1 | 2 | 21 | 3 | 1 | 1 | 1 | 2 | 1 |
| 1 | 2 | 22 | 1 | 1 | 1 | 2 | 2 | 2 |
| 1 | 2 | 22 | 3 | 1 | 2 | 1 | 2 | 2 |
| 1 | 2 | 21 | 4 | 2 | 2 | 1 | 2 | 2 |
