# Supplementary material for: Effects of virtual reality-based disaster simulation education on nursing students
Source: PLoS One. 2025 Oct 7;20(10):e0329563. doi: 10.1371/journal.pone.0329563 (PMC12503233; doi:10.1371/journal.pone.0329563)
Supplement: S2 Table — (DOCX) [file pone.0329563.s002.docx]

S2 Table. Variables dataset of participants

| Group | Disaster triage accuracy | | Disaster triage time required | | Disaster triage confidence | | Learning immersion | | Cognitive flexibility | |
| --- | --- | --- | --- | --- | --- | --- | --- | --- | --- | --- |
|  | Pre-test | Post-test | Pre-test | Post-test | Pre-test | Post-test | Pre-test | Post-test | Pre-test | Post-test |
| 2  2  2  2  2  2  2  2  2  2  2  2  2  2  2  2  2  2  2  2  2  2  2  2  2  2  2  2  2  2  2  2  2  2  1  1  1  1  1  1  1  1  1  1  1  1  1  1  1  1  1  1  1  1  1  1  1  1  1  1  1  1  1  1  1  1  1 | 2  2  3  2  2  2  3  1  3  2  3  1  3  1  2  2  2  2  2  2  2  2  3  2  3  2  3  2  3  3  2  2  3  3  2  3  2  3  2  3  2  3  2  2  3  2  3  1  2  3  3  2  3  2  3  2  2  1  3  2  3  2  2  2  2  3  2 | 3  3  3  3  3  3  3  2  3  3  3  3  3  2  3  3  3  2  3  3  3  3  3  3  3  3  3  3  3  3  3  3  4  3  4  4  4  3  4  3  4  4  4  4  4  4  4  4  4  4  4  3  4  4  4  4  5  4  4  4  5  4  4  5  4  4  5 | 91  94  96  90  91  106  96  99  83  82  79  74  96  90  91  92  98  89  90  89  86  91  95  96  109  94  76  96  90  91  106  99  89  91  88  80  91  91  99  96  109  94  69  96  90  89  81  82  98  96  110  94  90  94  96  90  75  106  97  99  84  71  79  92  106  99  89 | 79  75  87  84  85  87  82  77  79  84  72  79  82  77  80  85  72  76  70  78  73  73  83  70  76  78  75  77  82  67  82  70  67  74  79  68  74  79  70  69  77  73  68  66  63  68  64  73  81  79  80  82  77  72  75  80  67  73  67  75  70  70  79  68  67  71  72 | 7  4  5  5  3  5  6  4  6  6  6  5  7  4  5  4  6  5  7  8  7  6  5  6  6  3  3  5  4  5  4  5  5  4  4  4  3  6  7  6  5  6  5  7  8  7  4  6  4  7  6  5  7  5  7  6  5  6  7  5  5  4  6  5  6  3  5 | 7  4  5  5  6  7  6  7  7  5  6  7  5  5  6  7  6  6  7  4  5  5  6  7  6  7  7  5  6  7  5  5  6  7  8  8  9  8  7  8  9  8  8  8  6  6  6  7  8  8  9  8  7  8  9  6  6  7  5  5  6  7  8  8  8  9  9 | 3.5  3.5  3.7  3.7  3.5  3.3  3.8  3.8  3.6  3.6  3.9  3.6  3.3  3.8  3.7  3.7  3.6  3.9  3.5  3.4  3.7  3.3  3.5  3.5  3.0  4.1  3.6  4.0  3.5  4.1  3.5  3.6  3.4  4.3  3.2  3.4  3.6  3.5  3.4  3.3  3.6  3.5  3.4  3.0  3.6  3.5  3.9  3.4  3.5  3.8  3.6  3.9  3.6  4.2  3.5  3.7  3.8  3.9  4.5  3.3  3.2  3.1  3.2  3.2  3.3  3.4  2.9 | 3.8  4.2  3.9  3.9  3.9  3.9  3.4  4.0  3.6  4.0  4.0  4.2  3.6  4.0  4.0  4.2  3.4  4.1  3.4  3.5  4.0  3.6  4.3  4.2  3.5  3.6  3.2  3.5  3.3  4.0  4.4  3.6  4.7  3.9  4.8  3.6  4.4  4.0  4.3  4.0  4.3  4.1  4.4  4.4  4.0  4.5  4.2  4.7  4.4  4.5  4.6  4.4  4.4  4.4  4.7  4.1  4.3  4.2  4.5  4.3  4.4  3.8  4.3  4.1  4.9  4.6  4.8 | 3.53  3.84  3.63  3.68  3.42  4.11  3.79  3.47  3.37  3.32  3.89  3.79  3.21  3.74  3.16  3.16  3.21  3.79  3.11  3.74  3.37  3.68  3.42  4.16  4.11  3.47  3.37  3.42  3.37  3.74  3.11  3.11  3.74  3.11  3.74  3.11  3.74  3.11  3.47  3.74  3.05  3.63  3.05  3.37  3.47  3.47  3.74  3.16  3.74  3.16  3.74  3.21  3.42  3.74  3.47  3.74  3.21  3.68  3.42  3.95  3.89  3.68  3.37  3.42  4.16  3.74  3.21 | 3.63  3.63  3.42  3.63  3.16  3.63  3.32  3.37  3.21  2.95  3.74  3.53  3.05  3.37  3.26  3.05  3.37  3.11  3.42  3.11  3.58  3.11  3.53  3.11  3.11  3.05  3.42  3.11  3.11  3.89  3.53  3.05  3.68  3.37  4.16  3.32  3.74  3.89  3.37  3.79  3.74  3.89  3.79  3.79  3.63  3.74  3.53  3.74  3.21  4.16  3.84  3.89  3.63  3.84  3.47  3.89  4.11  4.21  3.68  3.79  3.74  3.79  3.95  3.95  3.89  3.95  3.89 |
